# Feeding Practices of Children in an Urban Slum of Kolkata

**DOI:** 10.4103/0970-0218.58402

**Published:** 2009-10

**Authors:** Sima Roy, Aparajita Dasgupta, Bobby Pal

**Affiliations:** Department of Community Medicine, IPGME&R, Kolkata, India; 1 Department of Preventive and Social Medicine, All India Institute of Hygiene and Public Health, Kolkata, India

## Introduction

Good nutrition forms the basis for good health of a child. Nutrition is required for a child to grow, develop, stay active, and to reach adulthood as well.(1)

Exclusive breastfeeding (EBF) is recommended as the optimum method of feeding for the first 6 months of life and semi-solid foods are to be introduced after 6 months while continuing breastfeeding to meet the physiological requirements of the infants.(2)

Studies(3) have reported that the practices of early introduction of top feeds and late introduction of semi-solids are widely prevalent, more so in urban slums.

Studies by the Nutrition Foundation of India (NFI) in urban slums of three major cities (Mumbai, Kolkata, and Chennai) revealed serious erosion of breastfeeding practices. Other studies from urban slums repeatedly documented that exclusive breastfeeding was practiced in only 30-40% of infants, colostrum was discarded in upto 90%, use of prelacteal feeds was almost universal, use of feeding bottles, animal milk, and commercial milk formulae was very common. Also it was found that the introduction of complementary foods is markedly delayed and the foods lack the consistency, energy density and are fed in inadequate amounts and in unhygienic ways.(3)

With this background, the study had been conducted to assess the feeding practices of the children in an urban slum and to determine the factors influencing it, if any.

## Materials and Methods

A cross-sectional study was conducted in the field practice area of the Urban Health Centre, Chetla, which is an integral part of the All India Institute of Hygiene and Public Health, Kolkata. Out of the many slums served by the health centre, a slum was chosen by simple random sampling. From the family folders maintained by the health centre, it was found that 242 children in that slum were in the age group of 6 months to 2 years. Owing to resource (time and manpower) constraints, only 50% were studied, so the sample turned out to be 121. The required number of children were selected by simple random sampling from the family folder, which was the sampling frame for the children under study,. Data was collected by interviewing the mothers with a questionnaire schedule. Altogether, information about 120 children could be collected.

## Results

72.5% (87/120) were Hindus, 25.8% (31/120) were Muslims, and 1.7% (2/120) belonged to other religions. 65.9% (79/120) were from nuclear families. Mothers of 81.6% (98/120) were literate and of 69.1% (83/120) were housewives. 41.67% (50/120) of the children belonged to families whose per capita income per month was less than Rs. 500.

A total of 93.33% (112/120) of the children were delivered at health facilities and the rest at home. 29.16% (35/120) received prelacteal feed [Table 1] in the form of water, infant milk formula, cow milk and honey. Mothers of 41.66% (50/120) of the children were informed about EBF and it was obtained mostly from the health facility (56.67% i.e. 68/120). The others were informed by family members and peer groups. Prelacteal feeding was more prevalent among mothers who were not informed about EBF and the relationship was statistically significant [Table 2]. Most of the children (76.67% i.e. 92/120) received breast milk within 24 h. 90% (108/120) were fed with colostrums. 28.33% (34/120) received exclusive breast feeding for 6 months [Table 1]. EBF was less in literate mothers and the relationship was statistically significant [Table 2]. Inadequate milk production is the most common reason for not giving EBF, which is about 62.79% (54/86). Rest were due to lack of information, prematurity, illness of mother, and the summer season. 71.66% (86/120) were given complementary feeding at 6 months [Table 1]. Advice for timely introduction of complementary feeding was obtained from the health facility, guardian and peer groups. Common foods used were rice, dal, mashed potato, suji, cerelac etc.

**Table 1:** Feeding practices of the children (*n* = 120)

Practices checked	Number	%
Initiation of breastfeeding		
Within 1 h	20	16.67
1-24 h	72	60
After 24 h	28	23.33
Colostrum feeding		
Fed	108	90
Not fed	12	10
Prelacteal feeding		
Given	35	29.16
Not given	85	70.84
Type of breastfeeding		
Exclusive breastfeeding	34	28.33
Not exclusively breastfed	86	71.67
Time of initiation of complementary feeding		
Before 6 months	15	12.50
At 6 months	86	71.66
After 6 months	19	15.84

**Table 2:** Relationship between feeding pattern and certain social variables (*n* = 120)

	Mother literate	Mother illiterate	Total
Exclusively breastfed	24	10	34
Not exclusively breastfed	74	12	86
Total	98	22	120

*X*^2^= 3.89, df = 1, *P* = <.05			

	**Informed about EBF**	**Not informed about EBF**	**Total**

Prelacteal feeding given	6	29	35
Prelacteal feeding not given	44	41	85
Total	50	70	120

*X*^2^ = 12.39, df = 1, *P* = <.05

## Discussion

The urban poor constitute the fastest growing section of the population with millions of babies being born annually. Feeding practices of children among urban poor is far from satisfactory which leads to various conditions of ill health and malnutrition.(4)

It was observed that 29.16% received prelacteal feed. In an urban slum of Mumbai, Kulkarni(5) found that prelacteal feeding was about 36.1%, while in a Chandigarh slum, it was to the extent of 40%.(6)

Most of children (76.67%) received breast milk within 24 h. Rate of EBF for 6 months was 28.33%, which was only 7.8% as observed by Tiwari(7). Khokhar(8) however found that it was 35.2% in an urban slum of Delhi. EBF was less among literate mothers. The finding is based on a small sample. Further research is needed on a large sample to validate the finding. Mothers of 41.66% children were informed about EBF and it was obtained mostly from the health facility. Tiwari(7) found that only 3.8% of their study-population was informed about EBF. In the present study, 90% were fed with colostrums. Percentage of colostrum-feeding varied. Kulkarni(5) and Khokhar(8) found it to be more than 50% while Tiwari(7) and Kumar(6) found that most were deprived of the colostrums. Insufficient breast milk was the most common cause of discontinuation of breastfeeding as was also observed by Kulkarni.(5) 71.66% were given complementary feeding at 6 months which was also reported by Kulkarni(5) (82.5%).

Infant feeding practices in the community are strongly influenced by what people know, think, and believe about these issues. They are also strongly affected by social circumstances, economic factors, and other forces beyond an individual's intention and ideas.(9) Effective communication for behavioral change should therefore be the prime objective for ensuring optimal infant feeding.
